# Optimal Deployment of Vector Sensor Nodes in Underwater Acoustic Sensor Networks

**DOI:** 10.3390/s19132885

**Published:** 2019-06-29

**Authors:** Sunhyo Kim, Jee Woong Choi

**Affiliations:** 1 Maritime Security Research Center, Korea Institute of Ocean Science and Technology, Busan 49111, Korea; 2 Department of Marine Science and Convergence Engineering, Hanyang University-ERICA, Ansan, Gyeonggi-do, Seoul 15588, Korea

**Keywords:** underwater acoustic sensor network, optimal deployment, vector sensor node, particle velocity, communication performance surface

## Abstract

Underwater acoustic sensor networks have recently attracted considerable attention as demands on the Internet of Underwater Things (IoUT) increase. In terms of efficiency, it is important to achieve the maximum communication coverage using a limited number of sensor nodes while maintaining communication connectivity. In 2017, Kim and Choi proposed a new deployment algorithm using the communication performance surface, which is a geospatial information map representing the underwater acoustic communication performance of a targeted underwater area. In that work, each sensor node was a vertically separated hydrophone array, which measures acoustic pressure (a scalar quantity). Although an array receiver is an effective system to eliminate inter-symbol interference caused by multipath channel impulse responses in underwater communication environments, a large-scale receiver system degrades the spatial efficiency. In this paper, single-vector sensors measuring the particle velocity are used as underwater sensor nodes. A single-vector sensor can be considered to be a single-input multiple-output communication system because it measures the three directional components of particle velocity. Our simulation results show that the optimal deployment obtained using single-vector sensor nodes is more effective than that obtained using a hydrophone (three-channel vertical-pressure sensor) array.

## 1. Introduction

Underwater acoustic sensor networks (UWASNs) are widely used in several applications such as target detection and tracking, ocean pollution monitoring, and various ocean data collection techniques [1,2,3,4]. The effectiveness of a UWASN can be evaluated in terms of the connectivity among the sensor nodes deployed in a targeted area, as well as the coverage rate. It is hard to precisely deploy numerous sensor nodes in underwater environments, and these nodes are also very expensive. Therefore, in terms of efficiency, it is important to minimize the number of nodes deployed in a targeted area.

Several approaches have been proposed to determine the optimal positions of sensor nodes in UWASNs [5,6,7]. However, most attempts have assumed that the communication performance of every node deployed at the target underwater area is the same without considering variations in the ocean environment. Acoustic waves propagating through underwater channels undergo multiple interactions with the ocean boundaries such as the sea surface and seabed, which cause severe time delays, resulting in inter-symbol interference (ISI) [8]. ISI causes issues related to communication demodulation. In addition, underwater communication is characterized by a time-varying channel, which produces large Doppler spreading and a short coherence time [9,10,11]. In addition, marine organisms such as barnacles and algae might build up on a transducer as time goes on, which can block the communication signals, causing data corruption. For these reasons, underwater acoustic modems should be energy-efficient and resistant to biofouling [12].

Recently, Kim and Choi [13] suggested a new algorithm to maximize communication coverage using a limited number of sensor nodes in shallow water areas. For this, we implemented a communication performance surface (PS) in our algorithm. The communication PS is a geospatial map displaying the communication performance information of the targeted area. The PS was obtained based on channel impulse responses predicted by a BELLHOP model [14] for grid points in the targeted area. The BELLHOP model is a highly efficient acoustic ray-tracing model for predicting acoustic pressure fields in ocean environments. 

Based on the PS, a virtual force-particle swarm optimization (VFPSO) algorithm was used to determine the optimal positions of the sensor nodes. We assumed that each sensor node was a vertical line array composed of vertically separated receiver elements to improve the communication performance. The array processing techniques can be used to enhance the communication performance by alleviating the effect of ISI [15,16]. However, such a large-scale array system degrades the spatial efficiency in a UWASN.

In recent years, the propagation properties of acoustic vector quantities such as acceleration or acoustic particle velocity have been studied [17,18,19]. Vector sensors have been applied in several areas, including target detection and tracking, noise reduction, and acoustic communication [20,21,22,23,24]. A vector sensor measures the three directional components of the vector quantities, and accordingly, a diversity gain can be achieved even when a single-vector sensor is used. In this paper, a vector sensor measuring the particle velocity is used as a sensor node of a UWASN, with the aim of improving the communication performance and the spatial efficiency. The outputs of the vector sensor are considered to be the outputs of a single-input multiple-output (SIMO) communication system [22,23,24]. The optimal algorithm proposed in our previous paper [13] is then applied to obtain the optimal deployment scheme for the single-vector sensor nodes.

This paper is organized as follows. Section 2 provides a short summary of previous research and describes the algorithm to calculate the communication PS of the single-vector sensor nodes. Section 3 describes the optimal deployment simulation for single-vector sensor nodes and compares the results to that for the hydrophone (three-channel pressure sensor) array. Finally, Section 4 provides a summary and conclusion.

## 2. Communication Performance Based on Single-Vector Sensors

### 2.1. Summary of Previous Research

Recently, Kim and Choi [13] suggested a methodology to maximize communication coverage while maintaining connectivity among the sensor nodes for a given number of nodes. It was assumed in most previous studies that the communication ranges of all underwater sensor nodes were identical. In contrast, the algorithm proposed by Kim and Choi finds the best locations of the sensor nodes based on a communication PS, which represents the spatial distribution of the relative performances in an underwater communication system. The algorithm is composed of four subcategories: (1) underwater acoustic channel modeling, (2) communication performance estimation, (3) the communication PS construction, and (4) optimal nodal placement. In their study, the targeted area in the East Sea of Korea was 22 km × 22 km and was divided into 100 grid points. The underwater acoustic channel impulse responses, as a function of the distance between the source and receiver, were simulated using the BELLHOP ray-tracing model with eight azimuthal angles for each grid point in the targeted area. The acoustic parameters for the targeted underwater area (such as bathymetry, water sound speed profile, and geoacoustic parameters for surficial sediments) are required to produce accurate BELLHOP model outputs.

We assumed a SIMO system consisting of sensor nodes in which each receiver was a 3-channel vertical line array. The communication signals received at every sensor array were simulated through convolution of the original binary phase-shift keying (BPSK) communication sequence with the estimated channel impulse responses. Then, the signals were decoded using multichannel combining and adaptive decision feedback equalization (DFE), and then the bit error rate (BER) was estimated. The communication range was defined as the range corresponding to a BER of 2%, which was used as a tolerance criterion for the sensor network. The communication ranges were averaged for the eight azimuthal angles and then interpolated for all grid points to create the communication PS. Lastly, based on the PS map, the VFPSO method [25] was applied to obtain the optimal deployment scheme of the underwater sensor nodes. The details of the optimal deployment procedure are given in Reference [13].

### 2.2. Underwater Acoustic Communication Using Single-Vector Sensors

**Figure 1:**
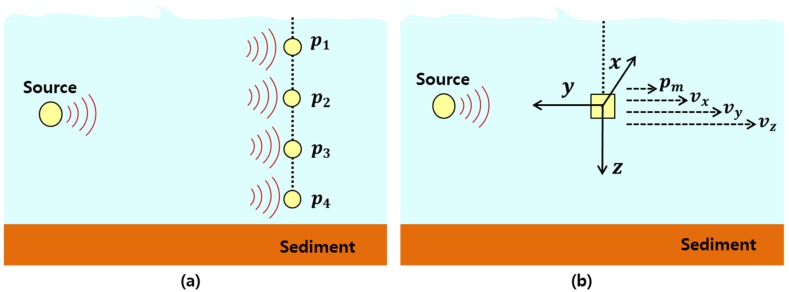
Communication system layouts. (**a**) Vertical-pressure sensor array, (**b**) single-vector sensor.

An acoustic vector sensor measures the particle motion produced by acoustic waves, which is a vector quantity described using the displacement, particle velocity, and acceleration, whereas a hydrophone measures the acoustic pressure, which is a scalar quantity. Most previous studies in underwater acoustic communication used hydrophone arrays to receive the communication signals [15,16], but this approach has poor spatial efficiency (Figure 1a). In contrast, a vector sensor has good spatial efficiency because it measures the three directional vector quantities as well as the pressure change at a single point. Accordingly, it can be used as a SIMO system, as shown in Figure 1b [22,23,24].

**Figure 2:**
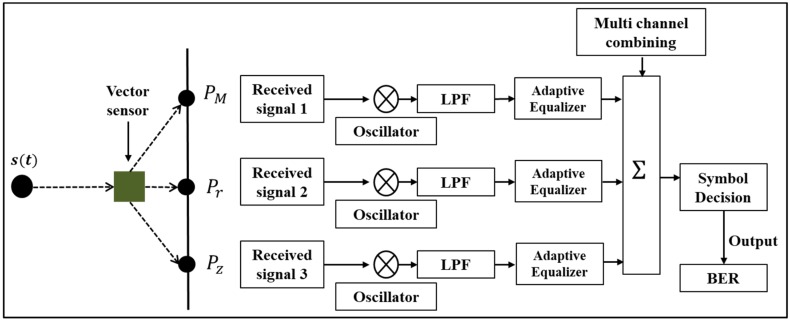
Block diagram of a single-input multiple-output (SIMO) system using a single-vector sensor. $P_{M}$ is an acoustic pressure. $P_{r}$ and $P_{z}$ are the horizontal and vertical components of the pressure equivalent particle velocities, respectively.

In this paper, it is assumed for convenience that the vector sensor measures particle velocity and pressure simultaneously in two-dimensional (2-D) coordinates (i.e., horizontal and vertical components). Thus, the vector sensor can be considered a one-by-three SIMO system for underwater acoustic communication. Figure 2 shows a block diagram of a vector sensor communication system for decoding the communication data. To recover the baseband waveform, the three components of the communication signal are multiplied by $e^{-i\omega t}$, where *ω* is the angular frequency. Then the rest of the process is the same as that in Reference [13], i.e., the baseband data are passed through a low pass filter and an adaptive DFE with a recursive least-squares (RLS) algorithm [26]. Then the data from multi-channels are combined to improve the signal-to-noise ratio (SNR) by removing residual ISI [15,26,27]. The BER performance is finally evaluated. See [24] for a detailed description of vector sensor communication using particle velocity and pressure signals.

**Figure 3:**
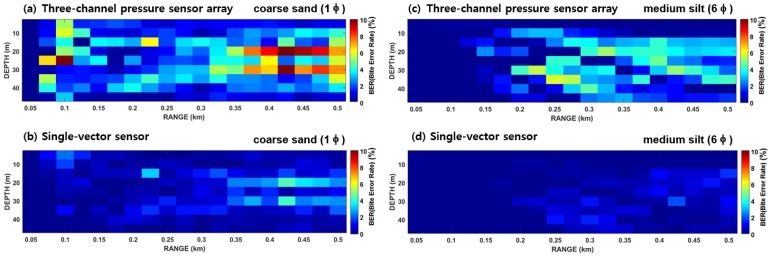
Communication performance (BER estimates) as a function of receiver position. Left and right plots are for coarse sandy and silty bottoms, respectively. Upper and lower plots correspond to the performance results for a three-channel pressure sensor array and a single-vector sensor, respectively.

Figure 3 shows the communication performance (BER) as a function of receiver position, estimated from a three-channel vertical-pressure sensor array and a single-vector sensor in a 2-D underwater environment with acoustically hard and soft bottoms. For easier comparisons, a Pekeris waveguide with a water depth of 50 m and a water sound speed of 1500 m/s was assumed. In addition, it was assumed that the sound source was at a depth of 25 m and transmitted a BPSK communication sequence with a 1 kbps bit rate and a 10 kHz carrier frequency. The BER performance was obtained as a function of receiver position by varying the receiver depth from 5 m to 45 m at 5-m intervals, and by varying the source-receiver range from 75 m to 500 m at 25-m intervals. In the pressure sensor array, the element spacing was 1.5 m, which corresponded to 10 times the wavelength. The communication signals received at each hydrophone channel were simulated using the method described in Reference [13]. The underwater acoustic channel response at each channel point was predicted by the ray-based BELLHOP propagation model and was convolved with a representation of the BPSK sequence.

A finite difference approximation method [28] was used to simulate the particle velocities received at the vector sensor. The particle velocity v(t) can be estimated by the time integral of the pressure gradient. The pressure gradient can be approximately estimated using two pressure signals received at two pressure sensors placed close together. The particle velocity is given by [24]:

(1) $$v\left(t\right)=\;\frac{1}{\rho_{0}}\;\overset{t}{\underset{0}{\int }}\frac{P_{2}\left(\tau \right)-\;P_{1}\left(\tau \right)}{d}d\tau$$

where $P_{1}$ and $P_{2}$ are the acoustic pressure signals at the two adjacent sensors, d is the sensor spacing between two sensors, $\rho_{0}$ is the water density, and τ is the time variable. The acoustic pressure is equivalent to the particle velocity in a particular direction multiplied by the acoustic impedance, which is $\rho_{0}c$, where c is water sound speed. Then, the acoustic pressure $P_{M}$ at the center between two adjacent sensors, and the horizontal and vertical components ($P_{r}$, $P_{z})$ of the pressure equivalent particle velocities are calculated by:

(2) $$P_{M}\left(t\right)=\;\frac{P_{1}\left(t\right)+P_{2}\left(t\right)}{2},\;\;P_{r}\left(t\right)=\;-\rho_{0}c\;v_{r}\left(t\right),\;\;P_{z}\left(t\right)=\;-\rho_{0}c\;v_{z}\left(t\right)$$

where $v_{r}$ and $v_{z}$ are the horizontal and vertical components of the particle velocities, respectively. For the simulation of particle velocities, two points, which were 3 cm apart both horizontally and vertically, were selected, and their center was considered to be the receiver point. Then the pressure channel impulse responses for these two adjacent points were predicted by the BELLHOP propagation model, and finally, $P_{M}$, $P_{r}$, and $P_{z}$ were calculated by Equation (2). The distance between the two adjacent points was equivalent to one fifth of the acoustic wavelength.ɸ.

For the comparison of BER performances shown in Figure 3, two different kinds of bottom types were considered. The hard bottom was a coarse sandy sediment with a mean grain size of 1.0 ɸ and the soft bottom was a silty sediment with a mean grain size of 6.0 ɸ where ɸ = $-\log_{2}$(d/$d_{0}$), d and $d_{0}$ are the grain diameter in millimeters and a reference length of 1 mm, respectively]. In general, an acoustically hard bottom causes severe ISI due to strong reflection from the water-bottom interface, whereas in case of a soft bottom, the number of dominant multipaths is limited due to a large bottom loss. Overall, the BER performances obtained by the single-vector sensor outperformed those obtained by the vertical-pressure sensor array in both the hard- and soft-bottom cases (Figure 3). In addition, the performances in silty sediment were better than those in sandy sediment due to the effect of ISI.

### 2.3. Communication PS Based on a Single-Vector Sensor

A method of optimal deployment using a communication PS has been suggested in Reference [13]. A communication PS shows the spatial distribution of the communication performance [29]. Therefore, if a communication PS is used in the process to search the optimal deployment positions, the rapid computation of the search process becomes possible. In this paper, the same targeted area specified in Reference [13] was used to evaluate the optimal deployment algorithm of the single-vector sensor nodes and to compare it with the results obtained using the pressure sensor array. The size of the targeted area was again 484 km^2^, of which the western boundary was ~10 km away from the east coast of Korea. A detailed description of the ocean environment in the targeted area, including bathymetry, water sound speed profiles, and sediment geoacoustic properties, is provided in Reference [13]. The targeted area was split up into 100 grid points. The channel impulse responses as a function of the range for eight different azimuths (every 45° from 0° North) of each grid point were simulated using the BELLHOP propagation model. The environmental, communication, and geometrical parameters used to extract the communication PS are shown in Table 1.

**Figure 4:**
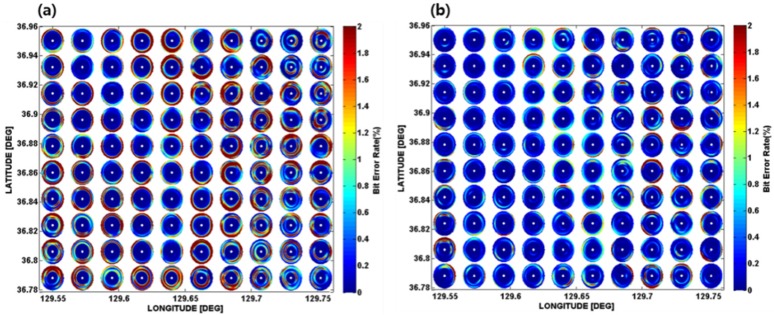
BER performance field for 100 grid points predicted for (**a**) the three-channel pressure sensor array, and (**b**) the single-vector sensor in the targeted area in February.

The simulated channel impulse responses were convolved with the original communication sequence to simulate the communication signals. Then, BER estimates were performed for the three-channel vertical-pressure sensor array and the single-vector sensor using the demodulation process shown, as shown in Figure 4 in Reference [13] and Figure 2 in this paper, respectively. Figure 4 shows the BER performances interpolated for the eight azimuths of each grid point for the two receiver systems. The results show that the single-vector sensor performed better than the three-channel vertical-pressure sensor array. Based on these results, the range corresponding to a BER of 2% was defined as the communication range, as in our previous work [13].

**Figure 5:**
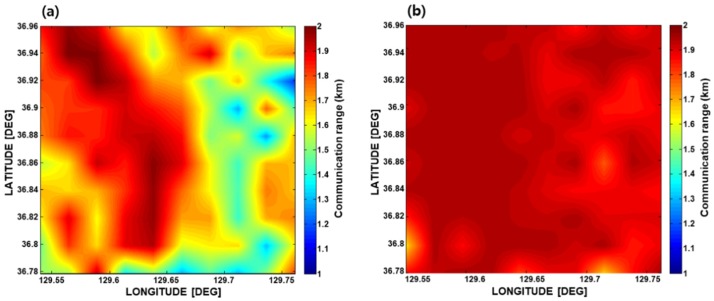
Communication PS simulated for the targeted area simulated for (**a**) the three-channel pressure sensor array, and (**b**) the single-vector sensor in the targeted area in February.

Then the communication ranges for the eight azimuths were averaged, and the mean value was defined as the communication radius for each grid point, assuming that the communication radius represents the communication performance for the grid point. Finally, the communication PS was obtained by an interpolation of the communication radii for every grid point. Figure 5a,b show the communication PS simulated for the three-channel pressure sensor array and for the single-vector sensor, respectively. The results also imply that the single-vector sensor has a better communication range compared with the three-channel pressure sensor array.

## 3. Optimal Deployment of Underwater Sensor Nodes

The optimization algorithm we used for sensor node deployment was the VFPSO [25], as used in Reference [13]. This algorithm is a hybrid method that combines the advantages of the virtual force algorithm (VFA) [30,31] and particle swarm optimization (PSO) [32,33], which was also used in Reference [13]. The PSO can find a global optimal position by comparing the particle position of the current generation to the experiences achieved at previous generations, and the VFA has an outstanding ability to adjust the distance between sensor nodes using attractive and repulsive forces. Therefore, in this case the VFPSO was suitable to search the optimal positions of sensor nodes where the communication coverage rate was maximized while ensuring that the connectivity of the sensor nodes was maintained. A detailed description of the VFPSO algorithm for optimal sensor node deployment is provided in Reference [13].

In this paper, one hundred sensor nodes were first randomly placed in the targeted area, and the VFPSO algorithm was then applied to the three-channel vertical-pressure sensor array and the single-vector sensor, based on the PS obtained in Section 2. The parameters used in the VFPSO algorithm for the optimal deployment simulation are given in Table 2.

**Figure 6:**
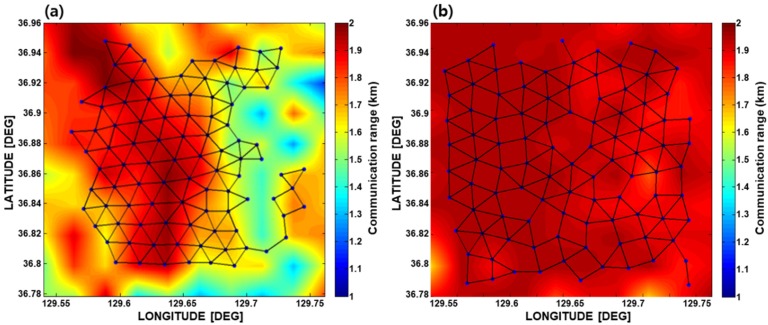
Results of the optimal deployment simulation using two different sensor node types: (**a**) the three-channel vertical-pressure sensor array, and (**b**) the single-vector sensor. The solid lines between the sensor nodes indicate their connectivity, which was estimated by comparing the communication range of each sensor node with the range from each sensor node to its neighboring nodes. In both cases, every sensor node was 100% connected.

Figure 6 shows one hundred sensor nodes deployed optimally in the targeted area, and their connectivity, which were displayed on their communication PS. The efficiency of the optimal deployment could then be investigated using the communication coverage rate, which was defined as the ratio of the area of communication coverage by the sensor nodes to that of the targeted area [13]. As a result, the communication coverage rate simulated using the single-vector sensor nodes was estimated to be 95.3%, whereas that of the three-channel pressure sensor nodes was 85.2%. This result was expected because the single-vector sensor had better communication performance compared to the three-channel pressure sensor array, as mentioned in Section 2.3.

## 4. Summary and Conclusions

Most previous studies on underwater acoustic communication used a multichannel underwater communication system with an array of spatially separated hydrophone receivers to increase the communication performance. Our previous paper [13] proposed a methodology of optimal deployment of underwater sensor nodes, which employed hydrophone arrays. Our algorithm used the communication PS, which can reflect the effects of ocean environmental variations on underwater communication. However, a large-sized receiver system compromises the spatial efficiency of a UWASN system.

This paper considers a single-vector sensor that measures particle velocities as an underwater sensor node in a UWASN system, instead of a hydrophone array measuring acoustic pressure, which is a scalar quantity. Since a vector sensor measures the three directional components of acoustic vector quantities, it can be used as a SIMO system. Therefore, diversity gain can be achieved during the decoding process of underwater acoustic communication, even with a single-vector sensor. Our method to determine the optimal deployment of sensor nodes (except for the difference of signal demodulation process owing to sensor type) was the same as in our previous work [13]. As a result, in our simulation, the performance of a single-vector sensor was better than that of the three-channel vertical-pressure sensor array. Specifically, the communication coverage rate obtained using one hundred single-vector sensors (which was 95.3%) was a ~10% improvement over that obtained using one hundred three-channel vertical-pressure sensor arrays (which was 85.2%). The simulation was further carried out and the results indicated that the communication coverage rate for one hundred three-channel hydrophone arrays could be achieved using 80 single-vector sensor nodes, with a coverage rate of 84.7%. In addition, when the simulation was repeated using one hundred single pressure sensor nodes, the coverage rate was estimated to be only 45.6%.

In this paper, we proposed an algorithm for determining the optimal locations of underwater sensor nodes under the assumption that each sensor node was composed of a single-vector sensor. If a vector sensor array is used as a sensor node, the spatial efficiency might be further improved, thus achieving better communication performance and larger communication coverage. However, both the advantages and disadvantages should be considered with respect to spatial efficiency and cost efficiency.

## Figures and Tables

**Table 1:** The parameters used for the communication PS simulation.

Ocean Environmental Parameters	Value	Communication Parameters	Value
**Month**	February	Symbol number	3500
**Longitude direction distance**	22 km	Symbol rate	1000 sps
**Latitude direction distance**	22 km	Pulse shaping	Root Raised Cosine filter
**Wind speed**	10 m/s	Equalizer	Adaptive DFE (RLS)
**Azimuth angle interval**	45°	BER criterion	2%
**Grid points**	100		
**Channel Modeling Parameters**		**Value**	
**Frequency**		10 kHz	
**Source level**		140 dB	
**Source depth**		2 m above the bottom	
**Three-channel vertical-pressure sensor array**	Sensor depth	0.5–3.5 m above the bottom	
	Element spacing	1.5 m (10 λ)	
**Single-vector sensor**	Sensor depth	2 m above the bottom	

**Table 2:** The parameters used in the simulation for optimal sensor node deployment.

Optimal Deployment Parameters	Value
Loop number	50
Sensor node number	100
Attractive force weight	0.01
Repulsive force weight	0.5
Acceleration weight	1
